# Classification of Dengue Fever Patients Based on Gene Expression Data Using Support Vector Machines

**DOI:** 10.1371/journal.pone.0011267

**Published:** 2010-06-23

**Authors:** Ana Lisa V. Gomes, Lawrence J. K. Wee, Asif M. Khan, Laura H. V. G. Gil, Ernesto T. A. Marques, Carlos E. Calzavara-Silva, Tin Wee Tan

**Affiliations:** 1 Department of Virology and Experimental Therapy, Aggeu Magalhães Research Center-CPqAM/FIOCRUZ, Recife, Brazil; 2 Department of Biochemistry, Yong Loo Lin School of Medicine, National University of Singapore, Singapore, Singapore; 3 Center for Vaccine Research, Department of Infectious Diseases and Microbiology, University of Pittsburgh, Pittsburgh, Pennsylvania, United States of America; 4 Department of Cellular and Molecular Immunology, Rene Rachou Research Center - CPqRR/FIOCRUZ, Belo Horizonte-MG, Brazil; Innsbruck Medical University, Austria

## Abstract

**Background:**

Symptomatic infection by dengue virus (DENV) can range from dengue fever (DF) to dengue haemorrhagic fever (DHF), however, the determinants of DF or DHF progression are not completely understood. It is hypothesised that host innate immune response factors are involved in modulating the disease outcome and the expression levels of genes involved in this response could be used as early prognostic markers for disease severity.

**Methodology/Principal Findings:**

mRNA expression levels of genes involved in DENV innate immune responses were measured using quantitative real time PCR (qPCR). Here, we present a novel application of the support vector machines (SVM) algorithm to analyze the expression pattern of 12 genes in peripheral blood mononuclear cells (PBMCs) of 28 dengue patients (13 DHF and 15 DF) during acute viral infection. The SVM model was trained using gene expression data of these genes and achieved the highest accuracy of ∼85% with leave-one-out cross-validation. Through selective removal of gene expression data from the SVM model, we have identified seven genes (MYD88, TLR7, TLR3, MDA5, IRF3, IFN-α and CLEC5A) that may be central in differentiating DF patients from DHF, with MYD88 and TLR7 observed to be the most important. Though the individual removal of expression data of five other genes had no impact on the overall accuracy, a significant combined role was observed when the SVM model of the two main genes (MYD88 and TLR7) was re-trained to include the five genes, increasing the overall accuracy to ∼96%.

**Conclusions/Significance:**

Here, we present a novel use of the SVM algorithm to classify DF and DHF patients, as well as to elucidate the significance of the various genes involved. It was observed that seven genes are critical in classifying DF and DHF patients: TLR3, MDA5, IRF3, IFN-α, CLEC5A, and the two most important MYD88 and TLR7. While these preliminary results are promising, further experimental investigation is necessary to validate their specific roles in dengue disease.

## Introduction

Dengue virus (DENV) is an emerging mosquito-borne pathogen that infects approximately 50–100 million people every year. The virus has an RNA genome and exists as four major serotypes (DENV1-4) that are phylogenetically distinct [1]. According to the World Health Organization (WHO) reports during the past 50 years, the incidence of dengue has increased 30-fold and it is estimated that 2.5 billion people live in endemic areas spread over 100 countries [2]. Symptomatic infection by DENV can range from a mild disease, dengue fever (DF), to a severe dengue haemorrhagic fever (DHF), which can culminate with dengue shock syndrome (DSS) and death. Although sequential dengue infection by distinct serotypes and antibody-mediated enhancement (ADE) are the two most studied risk factors for the development of the severe diseases, the key determinants of DF or DHF progression remain elusive [2]–[6].

During infection, the innate immune response plays an important role as the first line of defence and also in the shaping of the adaptive responses. One of the first steps of the innate responses is the recognition of pathogen-associated molecular patterns (PAMPs) [7]. The innate immune system identifies these patterns through pattern recognition receptors (PRRs) [8], such as the Toll-like receptors (TLRs) 3, 7, and 9 located at the cellular membranes, and also by cytoplasmic proteins, like the retinoic acid-inducible gene-I (RIGI) and melanoma differentiation-associated gene 5 (MDA5). Binding of viral molecules to these PRRs results in a downstream activation of a gene cascade, including MYD88 (an adapter protein that serves as an intermediate molecule), transcriptions factors (IRF3 and IRF7), and the expression of interferon (IFN) type I and II genes [7]. IFNs are produced by many types of cells and induce an antiviral state by up-regulating genes with direct and indirect antiviral functions [7]–[15]. While it is known that IFNs are the first line in the host defence against DENV infections [7], and that DENV inhibits the IFN signalling pathway [16], the actual relationship between the ability to block IFN signal and pathogenicity is not known. We previously postulated that the expression levels of the TLRs and the signal transduction molecules of IFN type I and II pathway would have a significant role in modulating the host IFN response to DENV and hence the disease outcome. The possible associations of this interplay with clinical outcomes appear complex, with indications that differential expression of different immunological pathways result in disparate clinical outcomes [17]. To investigate the possible role of the expression level of these genes at mRNA levels during DENV infection, it is necessary to design rational gene expression studies followed by careful analyses of gene expression patterns in well characterized dengue patients [18]. Towards this end, several groups have been studying disease susceptibility factors, via high throughput molecular typing or through association studies to identify disease associated candidate genes [2]. *In silico* approaches utilizing clinical data have been developed to improve dengue diagnosis and prognosis. Recently, our group developed a simple method to reliably differentiate primary and secondary acute dengue infections based on serological data (IgG ELISA) [19] and quickly identify individuals with a secondary dengue infection, which is considered as a risk factor. Nevertheless, only a small fraction of secondary infections develop DHF and the early detection of patients with risk of developing DHF is still not possible.

**Figure 1:**
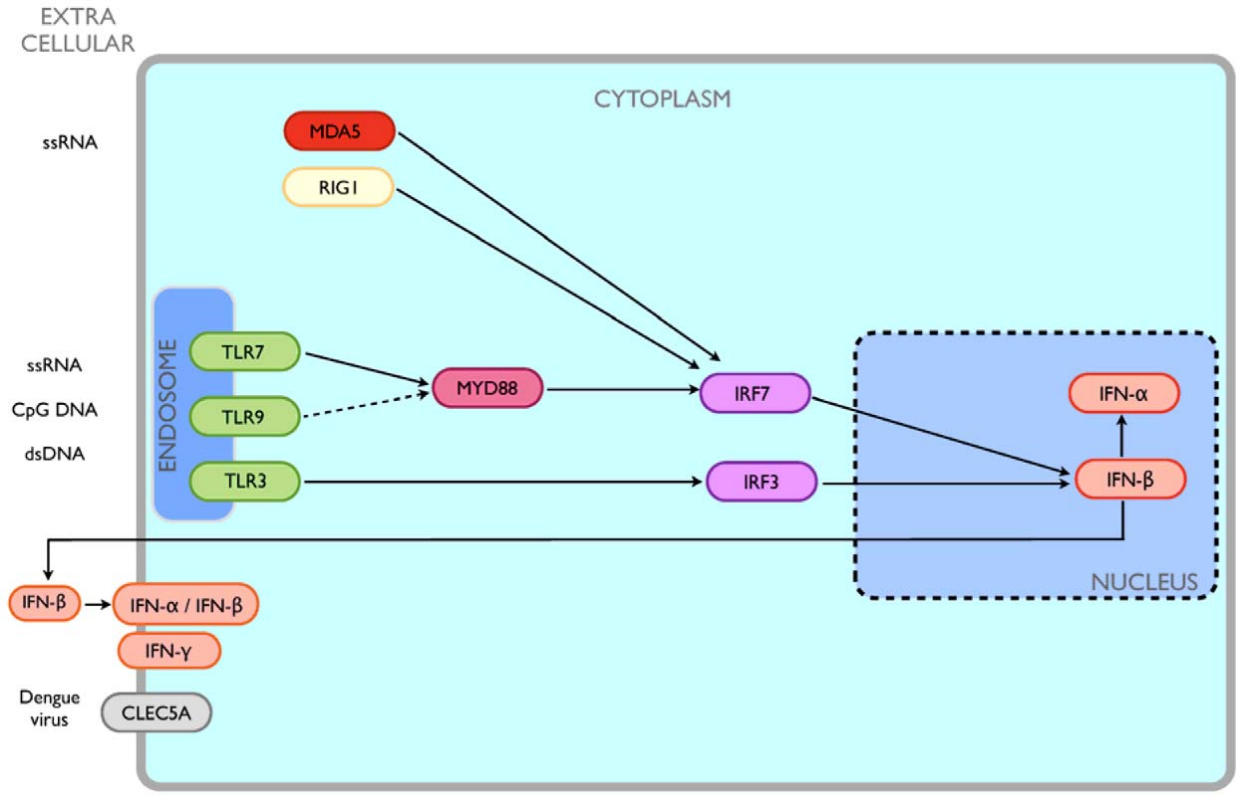
A schematic map depicting the interactions of the 12 proteins/genes studied herein, known or indicated to be relevant to the viral innate immune response pathway, including for dengue.

In this manuscript, we present a computational approach to classify DF and DHF patients based on mRNA expression data of 11 genes (MYD88, MDA5, TLR3, TLR7, TLR9, IRF3, IRF7, IFN-α, IFN-β, IFN-γ, and RIGI) involved in the innate immune response pathway using the support vector machines (SVM) algorithm. The significance of these genes in determining DF or DHF progression was also explored using this approach. Additionally, CLEC5A, a cell surface receptor, which is also involved in innate response, was included in the analysis as a reference marker because it has been proposed to be involved in the development of DHF [20]. A schematic map depicting the interactions of the 12 proteins/genes in the viral innate immune response pathway is provided in Figure 1. We selected these genes because i) they have been previously shown to have a significant fold-change in our cDNA microarray analysis [18] of genes expressed in patients with DF versus DHF, compared to non-dengue (ND) patients, or ii) they have been described to be relevant for hemorrhagic symptoms of dengue by others in the literature [7]–[10], [12], [17], [20]. The aim of this study was to help identify important genes or pathways of the innate immune responses involved in DF or DHF, which can then be used as markers.

SVM, introduced by Cortes and Vapnik [21], is a relatively new sub-branch of supervised learning methods, and has been shown to be highly effective for diverse computational biology applications [22]. Some example applications include prediction of secondary structure, quaternary structure, homology, domains, cleavage sites, protein-protein interaction, T-cell epitopes, classification and validation of cancer tissue samples and also microarray expression data. Our gene expression data from dengue disease patients provides an opportunity to better understand the relevance of the individual genes in the DENV infection pathway involving the innate immune response. Though the application of SVM as a classification tool for expression data is not new, its utility to analyse the role of genes of the innate immune response pathway in DENV has not been explored.

## Materials and Methods

### Ethics statement

All participants signed an informed consent. This study, related to gene expression of dengue patients and application of the SVM algorithm to the data is part of a bigger functional immunomics study in our lab and was reviewed and approved by ethics committee of Brazilian Ministry of Health CONEP: 4909; Process n° 25000.119007/2002-03; CEP: 32/09. In addition, the Johns Hopkins IRB also reviewed this study as protocol JHM-IRB-3: 03-08-27-01.

### Patient data

A total of 33 patients (5 ND, 15 DF and 13 DHF) were studied from the Recife metropolitan area, Brazil [23] and were classified following the WHO criteria [4]. Blood samples from patients enrolled in this study were collected in heparin vacutainer tubes (BD Vacutainer) and within 2 hours after the collection, peripheral blood mononuclear cell (PBMC) samples were separated by gradient density using Ficoll-Paque (Amersham Biosciences) and cryopreserved in 10% (v/v) Dimethyl sulfoxide (DMSO; Sigma-Aldrich) in inactivated fetal bovine sera (FBS; Hyclone). All samples were collected from patients between 3^rd^ and the 5^th^ day of onset of fever.

### Gene expression

RNA extraction was performed according to the manufacturer's manual for the Spin cell RNA mini kit (Invisorb). Total RNA was reverse transcribed to cDNA using SuperScript III First-strand Synthesis System for qPCR (Invitrogen) using random hexamer primers, according to the manufacturer's instructions. The expression of 12 genes related to viral innate immune response were quantified using qPCR based on 2∧-ΔΔCt method [24]. Expression values for each gene were normalized by use of beta-actin housekeeping gene, which were then quantified and related to ND cycle threshold (Ct) values (i.e. DF/ND ratio).

**Figure 2:**
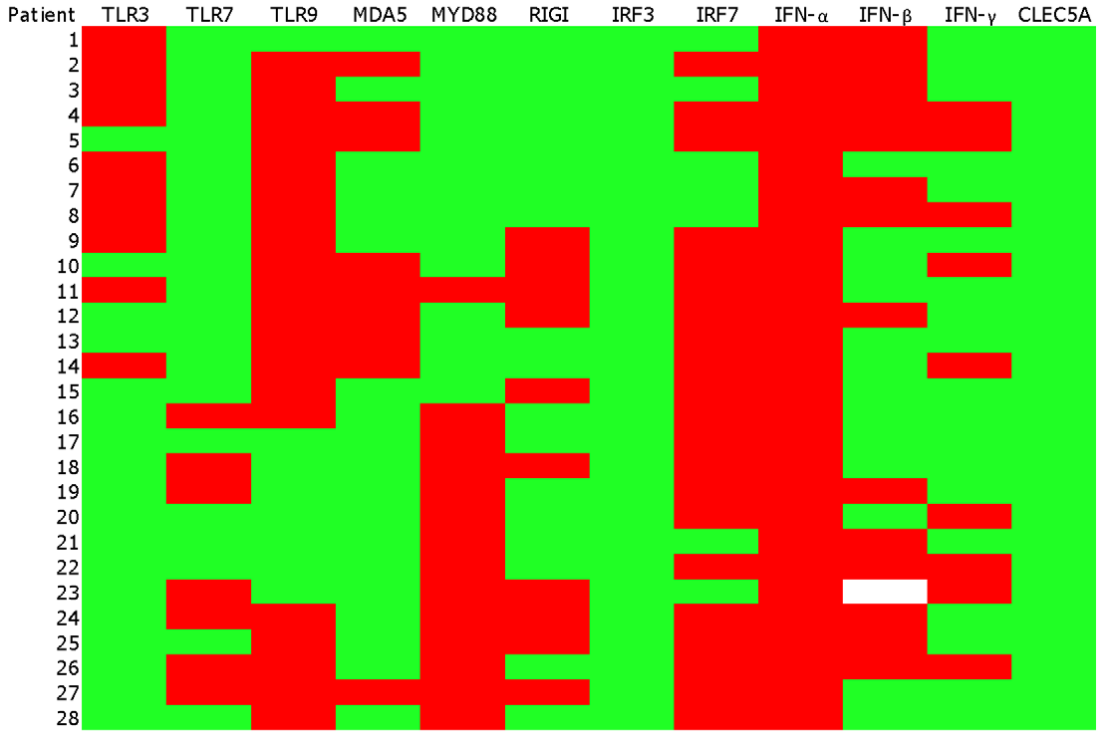
Heatmap for gene expression data of the 12 genes (columns) studied from the 28 patients (rows). The first 15 are DF patients, while the rest are DHF patients. The DF/ND and DHF/ND gene expression values from qPCR were used to create the heatmap. The colour shades are associated with the values in the cells: green for ratio of DF/ND and DHF/ND of <1 (down-regulated) and red for DF/ND and DHF/ND ratio of > = 1 (up-regulated). The gene expression data for IFN-β of one of the patients (23) was not available and therefore the vector attributes of this gene for the patient were represented as blank.

For each of the 12 genes tested, the DF/ND and DHF/ND ratio of qPCR experimental values were used to define the up-regulated and down-regulated states of the genes (Figure 2). DF/ND and DHF/ND ratio values of > = 1 were defined as up-regulated, while DF/ND and DHF/ND values of <1 were defined as down-regulated.

### Statistical analysis

Statistical analyses were performed on qPCR data using unpaired two-tailed Student's T test with Welch's correction due to the small sample size and because we assumed unequal variances in the data. All tests were done using the Prism software version 4.0a (www.graphpad.com).

### SVM implementation

The SVM algorithm is a machine learning technique developed based on the structural risk minimization principle of statistical learning theory (see [25] for details on the algorithm and [22] for details on its implementation, optimization, training and testing). Briefly, both positive and negative examples in a dataset were represented by feature vectors *x_i_* (*i* = 1, 2,…, *N*) with corresponding binary labels 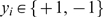. The SVM algorithm classifies the positive and negative examples by training a classifier which maps the input samples, using a kernel function in most cases, onto a high-dimensional space, and then seeks for a separating hyperplane that best differentiates the two classes with a maximal margin and a minimal error. The decision function for classification of unseen examples is given as:
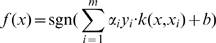
where 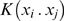 is the kernel function, and the parameters are determined by maximizing the following:
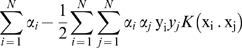
under the conditions,
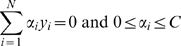
The variable *C* serves as the cost parameter that controls the trade-off between margin and classification error. As the efficacy of the SVM-based classification is dependent on the type of kernel used, we explored the use of various commonly used kernels (linear, sigmoid, polynomial and the radial basis function) on our datasets. We chose the radial basis function (RBF) kernel as it was found to be most effective (data not shown):
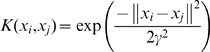
Two parameters are required to optimize the RBF kernel of the SVM classifier; γ, which determines the capacity of the RBF kernel and *C*, the regularization parameter.

### Vector encoding schemes

To encapsulate gene expression data in a format suitable for SVM training and testing, vectors based on the orthonormal encoding scheme were created. Each gene was designated as either “10” (for observation of up-regulation) or “01” (for observation of down-regulation). Therefore, the collective gene expressions observed in each patient was represented by a 24-dimension vector (12 genes×2 gene states: up- or down-regulated). Each of the 24-dimension vectors was labeled as either “1” for DF patients or “−1” for DHF patients, corresponding to positive and negative examples for SVM training, respectively. The first 15 rows in the input matrix corresponded to the gene expression data of the 15 DF patients analyzed herein, while the last 13 rows were of the DHF patients. The vector labels “1” and “−1” are in the first column of the matrix and the subsequent 12 columns of each row correspond to the gene expression states, in orthonormal encoding scheme, for the 12 genes studied. For example, the vector encoding “1 1∶1 4∶1…” represents data of a DF patient (1, first column) and the first two genes, TLR3 and TLR7, which are up-regulated and down-regulated, respectively.

It is noted that the gene expression data for IFN-β of one of the patients (23) was not available and therefore the vector attributes of this gene for the patient were represented as blank, and this notation was acceptable as our simulation studies of setting the gene as up- or down-regulated did not change the accuracy of the model.

### SVM model development

The SVM model was implemented using the freely downloadable LIBSVM package by Chang and Lin [26]. Here, the SVM model was used to classify the DF and DHF patients using gene expression data from the patients. We conducted leave-one-out cross-validation (LOOCV) on the patient gene expression dataset using various combinations of *γ* and *C*. In LOOCV, the patient vectors dataset was split into 28 training examples where one of the training examples was used as the test example while the others were used for training the SVM classifier. The trained classifier was tested on the test example. The process was repeated 28 times using different test and training examples each time, hence ensuring that all examples were included for both training and testing. SVM parameters *γ* and *C* were stepped through combinations of 0.01, 0.1, 1, 10, and 100 for *C*, and 0.1, 1, 10, 100, and 1000 for *γ* in a grid-based manner. To provide an indication of the overall performance of the model, the accuracy (AC), as given in the following equation, was calculated on the output of the LOOCV:
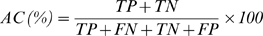
The values of *C* and *γ* that returned the optimal accuracy were noted. The corresponding accuracy value obtained was assigned as baseline.

The cross-validation procedure was repeated fifteen times under different conditions to analyse the individual and collective contributions of each gene expression data to DF/DHF classification. Specifically, in the first twelve trials, a different gene was removed from the vector dataset, while multiple genes were removed from the vector dataset in the last three trials. Corresponding changes to the baseline accuracy were measured for all cases (Table 1).

**Table 1:** Performance of SVM model for various combinations of genes tested. RBF kernel function (γ value = 1.0 and *C* value = 10) was utilized for model building.

Matrix conditions	Genes tested	Gene(s) removed	Accuracy (%)
1	MYD88, MDA5, TLR7, TLR9, IRF3, IRF7, CLEC5A, IFN-α, IFN-β, IFN-γ, and RIGI	TLR3	85.19
2	MYD88, MDA5, TLR3, TLR9, IRF3, IRF7, CLEC5A, IFN-α, IFN-β, IFN-γ, and RIGI	TLR7	81.48
3	MYD88, MDA5, TLR3, TLR7, IRF3, IRF7, CLEC5A, IFN-α, IFN-β, IFN-γ, and RIGI	TLR9	92.59
4	MYD88, TLR3, TLR7, TLR9, IRF3, IRF7, CLEC5A, IFN-α, IFN-β, IFN-γ, and RIGI	MDA5	85.19
5	MDA5, TLR3, TLR7, TLR9, IRF3, IRF7, CLEC5A, IFN-α, IFN-β, IFN-γ, and RIGI	MYD88	66.66
6	MYD88, MDA5, TLR3, TLR7, TLR9, IRF3, IRF7, CLEC5A, IFN-α, IFN-β, and IFN-γ	RIGI	92.59
7	MYD88, MDA5, TLR3, TLR7, TLR9, IRF7, CLEC5A, IFN-α, IFN-β, IFN-γ, and RIGI	IRF3	85.18
8	MYD88, MDA5, TLR3, TLR7, TLR9, IRF3, CLEC5A, IFN-α, IFN-β, IFN-γ, and RIGI	IRF7	88.88
9	MYD88, MDA5, TLR3, TLR7, TLR9, IRF3, IRF7, CLEC5A, IFN-β, IFN-γ, and RIGI	IFN-α	85.18
10	MYD88, MDA5, TLR3, TLR7, TLR9, IRF3, IRF7, CLEC5A, IFN-α, IFN-γ, and RIGI	IFN-β	92.59
11	MYD88, MDA5, TLR3, TLR7, TLR9, IRF3, IRF7, CLEC5A, IFN-α, IFN-β, and RIGI	IFN-γ	88.88
12	MYD88, MDA5, TLR3, TLR7, TLR9, IRF3, IRF7, IFN-α, IFN-β, IFN-γ, and RIGI	CLEC5A	85.18
13	MYD88, MDA5, TLR3, TLR7, IRF3, CLEC5A, and IFN-α	TLR9, RIGI, IRF7, IFN-β, and IFN-γ	96.26
14	MDA5, TLR3, TLR9, IRF3, IRF7, CLEC5A, IFN-α, IFN-β, IFN-γ, and RIGI	TLR7 and MYD88	62.96
15	MYD88 and TLR7	TLR3, TLR9, MDA5, RIGI, IRF3, IRF7, IFN-α, IFN-β, IFN-γ, and CLEC5A	88.88

## Results

**Figure 3:**
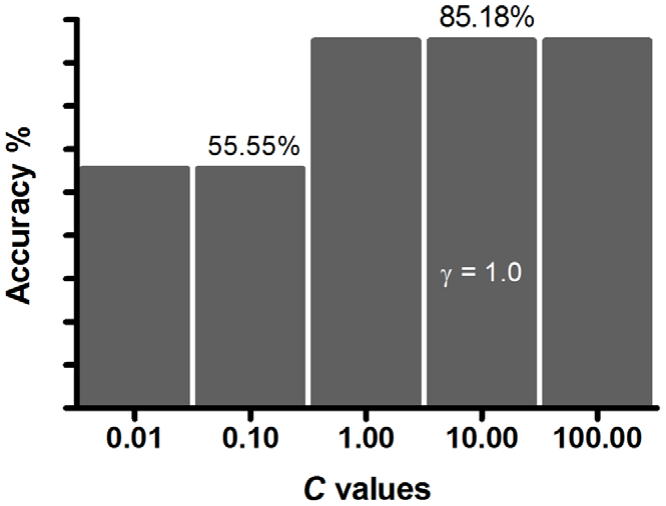
SVM optimization. Optimization of the parameters *C* and γ of the SVM kernel RBF: only *C* values of 0.01, 0.10, 1.0, 10.0 and 100.0, and γ value of 1.0 are shown.

A total of 12 genes (TLR3, TLR7, TLR9, RIGI, IRF3, IRF7, MYD88, CLEC5A, IFN-α, IFN-γ, IFN-β, and MDA5), were quantified in 28 dengue patients (13 DHF and 15 DF) using qPCR. The expression data was obtained from PBMCs of patients with DF and DHF during acute phase (between 3^rd^ and 5^th^ day of fever) before defervescence and any signs of vascular leakage. The gene expression data was used to explore associations of individual expression levels with DF and DHF using the SVM algorithm. We had selected the RBF kernel because it was shown to perform well on our datasets and it is also widely used for SVM classification in other related domains. The parameters (*C* and γ) of RBF were optimized by performing leave-one-out cross validation. The *C* and γ values that returned the highest accuracy of ∼85.18% were *C* of 1, 10 (selected for further work) or 100 and γ of 1.0 (Figure 3). Henceforth, this accuracy of ∼85.18% will be referred to as the baseline accuracy for the SVM model of the 12 genes.

### Influence of each gene to the accuracy of the SVM model

To define the contribution of each of the 12 genes on the determination of DF and DHF, selective removal of vector attributes corresponding to each specific gene expression data were carried out and the downstream effect on the baseline accuracy of the SVM model were measured. Vector attributes of genes removed from the input vectors that result in a significant reduction in the model accuracy are likely to be more relevant to determine the differences between the DF and DHF gene expression pattern. We postulated that genes with greater impact on the model accuracy would have a relatively greater importance defining the different innate immune response in DF and DHF patients.

**Figure 4:**
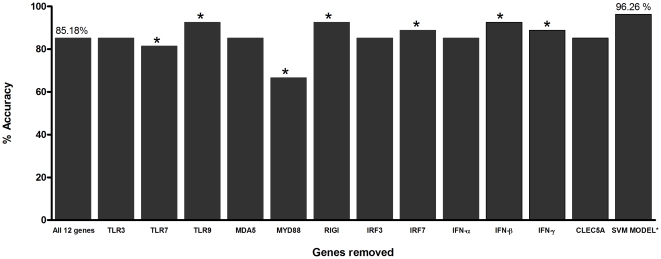
Influence of each gene to the accuracy of the baseline SVM model. The first bar represents the baseline accuracy of all the 12 genes (TLR3, TLR7, TLR9, MDA5, MYD88, RIGI, IRF3, IRF7, IFN-α, IFN-β, IFN-γ, and CLEC5A). The subsequent bars represent accuracy of datasets with only 11 genes, whereby vector attributes of one gene were removed at a time (the name of the gene removed is indicated). The last bar, SVM model refers to the seven genes (MYD88, TLR3, TLR7, MDA5, IRF3, IFN-α and CLEC5A) that returned optimum accuracy. The RBF kernel function of SVM with optimum parameter settings (*C* = 10 and γ = 1.0) were used for model building of each situation. * represents p<0.05 value compared with the 12 baseline gene set (the bar labelled “All 12 genes”).

The genes MYD88, TLR7, TLR3, MDA5, IRF3, IFN-α and CLEC5A were observed to be relevant in defining the distinction between DF from DHF expression patterns, and among these MYD88 and TLR7 were the two most important (Figure 4 and Table 1). Removal of MYD88 or TLR7 decreased the baseline accuracy to ∼66.66% and ∼81.48%, respectively. The individual removal of the genes TLR3, MDA5, IRF3, IFN-α, and CLEC5A from the input matrix dataset did not change the accuracy value obtained with all the 12 genes (∼85.18%). However, when these five genes were trained collectively with MYD88 and TLR7, the accuracy increased to ∼96.26% (Figure 4 and Table 1), indicating a subtle individual, but significant combined role of these five genes. According to our SVM results, the remaining five genes (TLR9, RIGI, IRF7, IFN-β and IFN-γ) showed negative effect to the classification of the patient types – individual removal of the five genes increased the accuracy (∼88.88 to ∼92.59%; Figure 4 and Table 1), suggesting that in the context of the genes selected and the disease outcome there is a lack of association between the mRNA levels of these five genes with the levels of the others. Taken together, the observations that the expression levels of signal transduction molecules had important effect in the model and the negative effects of IFN-β and IFN-γ suggest that impaired IFN signalling play important role in DHF.

## Discussion

In this study, we describe application of the SVM algorithm to classify DF and DHF patients based on the expression data of innate immune response pathway genes, to better understand their roles in the disease progression. SVM modelling of the expression patterns of the 12 genes in the dengue patients with different disease outcomes implicates the association of some of the genes with the severity of dengue symptoms. Our results suggest that MYD88 and TLR7 genes are relatively the most important for defining specific DF and DHF expression patterns. This finding is supported by experimental data that showed the utilization of the TLR7 receptor by DENV [5] and the direct influence of the virus on the expression of MYD88 [14]. In addition, it is clear from the gene expression data that the expression levels of TLR7 and MYD88 are significantly higher in DHF patients than in DF (Table 2). Based on these analyses, we posit that these two genes and possibly others in the TLR7 pathway are likely to be good candidates for further studies.

**Table 2:** Average gene expression ratios (DF/ND and DHF/ND) for the seven genes found to be important for the classification of DF and DHF patients.

Dengue clinical form	Genes						
	TLR7	MYD88	TLR3	MDA5	IRF3	IFN-α	CLEC5A
DF/ND	01	01	01	10	01	10	01
DHF/ND	10	10	01	01	01	10	01

The binary “01” means ratio of DF/ND and DHF/ND of <1 (down-regulated) and “10” means DF/ND and DHF/ND ratio of > = 1 (up-regulated).

Apart from MYD88 and TLR7, five other genes (TLR3, MDA5, IRF3, IFN-α, and CLEC5A) were observed to exhibit subtle individual, but significant collective role (Table 1). The significant involvement of the genes TLR3, IRF3 and MDA5 on the diagnostic specific expression pattern suggests that DENV likely interacts with more than one IFN activation pathway [7], [14]. CLEC5A, a cell surface receptor, has been reported to be differentially expressed between DF and DHF patients, suggesting involvement in the development of DHF [20]. However, in our study, the differences in mRNA expression levels of CLEC5A between DF and DHF patients were relatively less relevant than TLR7 and MYD88 (Table 2). This difference could be because of the different experimental approaches utilized, whereby our data was from clinical patients and theirs was an *in vitro* study. IFN-α, the other member of the five genes, is a well-established antivirus response factor and we observed over-expression for both DF and DHF patients. The five genes (TLR9, RIGI, IRF7, IFN-β and IFN-γ) that showed negative effect on the accuracy confounded the classification of DF and DHF patients. However, further investigation is necessary before we can eliminate their specific role in this regard. It is possible that the IFN-α is being produced but its signal is not being efficiently transduced.

Two important limitations of the present study include the small sample size of the patient data and the sampling bias. This may impact the interpretation of our results, which were based only on 28 patients from Brazil. Nevertheless, despite these limitations, we have shown the utility of the SVM method in deciphering the complex interplay of numerous genes involved in a biological pathway through gene expression data of patient samples. Moreover, the method is scalable as new patient sample data can be easily appended to the preliminary SVM model and reassessed. In addition, several novel machine learning algorithms have been reported by various groups to accurately select gene markers in expression studies [27]–[29]. As they are particularly effective for handling large datasets and feature dimensionality, it would be useful to explore these methods for DF and DHF classification as larger and more complex expression datasets become available.

Notably, the SVM model built on a large number of expression data can potentially be used to accurately classify the prognosis of patients with the benign form (DF) from those with the life-threatening (DHF) DENV disease. The lack of reliable classification tools to differentiate DF and DHF patients has often resulted in a large number of unnecessary and costly hospitalizations [6], [19]. Further, early diagnosis of DHF patients will represent better prognosis.

In summary, this is the first report of application of the SVM method to gene expression data from DF and DHF patients to better understand the role of the genes in DENV infection pathway. The results suggest important role of seven genes in classifying DF and DHF patients: TLR3, MDA5, IRF3, IFN-α, CLEC5A, and the two most important MYD88 and TLR7.
